# A massively parallel corpus: the Bible in 100 languages

**DOI:** 10.1007/s10579-014-9287-y

**Published:** 2014-11-19

**Authors:** Christos Christodouloupoulos, Mark Steedman

**Affiliations:** 1 Department of Computer Science, UIUC, 201 N. Goodwin Ave, Urbana, IL 61801 USA; 2 School of Informatics, University of Edinburgh, Edinburgh, UK

**Keywords:** Parallel corpus, Multilingual corpus, Comparative corpus linguistics

## Abstract

We describe the creation of a massively parallel corpus based on 100 translations of the Bible. We discuss some of the difficulties in acquiring and processing the raw material as well as the potential of the Bible as a corpus for natural language processing. Finally we present a statistical analysis of the corpora collected and a detailed comparison between the English translation and other English corpora.

## Introduction

Parallel corpora are a valuable resource for linguistic research and natural language processing (NLP) applications. One of the main uses of the latter kind is as training material for statistical machine translation (SMT), where large amounts of aligned data are standardly used to learn word alignment models between the lexica of two languages (for example, in the Giza++ system of Och and Ney 2003). Another interesting use of parallel corpora in NLP is *projected learning* of linguistic structure. In this approach, supervised data from a resource-rich language is used to guide the unsupervised learning algorithm in a target language. Although there are some techniques that do not require parallel texts (e.g. Cohen et al. 2011), the most successful models use sentence-aligned corpora (Yarowsky and Ngai 2001; Das and Petrov 2011).

Most parallel corpora exist in a small number of languages or in common languages pairs (e.g. the English-French *Hansards* corpus by Germann 2001). There are however, a few corpora that contain multiple languages: The Europarl corpus (Koehn 2005) contains parallel translations of European Parliament proceedings in 21 languages; the Joint Research Centre of the European Commission has released multiple corpora in more than 20 languages, including the sentence-aligned JRC-Acquis (22 languages, Steinberger et al. 2006) and the paragraph-aligned DGT-Acquis (23 languages); the InterCorp corpus (Čermák and Rosen 2012), a collection of texts in Czech and 27 other European languages. To our knowledge, the most multilingual corpus currently available is the OPUS collection Tiedemann 2012 which contains 90 languages in various parallel corpora. However, comparatively few of the possible language pairs are available with parallel text.^1^

In an attempt to access parallel material from as many and as diverse languages as possible, a very widely translated text is needed. In this work we will be following Resnik et al. (1999) in creating a massively parallel corpus based on Bible translations (cf. Abney and Bird 2010, 2011). According to United Bible Societies (2013) there are at least 2,527 translations of parts of the Bible and 475 full translations. These numbers exceed by far the translations of any other work of literature—according to Wikipedia (2013) the next most translated work of literature is ‘Pinocchio’ with 260 languages.

Resnik et al. (1999) used 13 different translations of the Bible; we will increase the number of languages to 100. By having 100 different languages on the same corpus we can get 4,950 unique language pairs^2^—although not all translations contain the entire Bible as we shall see later—making this by far the largest number of bitexts available: in comparison, DGT-Acquis contains 253 pairs; InterCorp, 351; and the OPUS collection contains 3,800 pairs (Tiedemann 2012), but not all pairs contain the same amount of text.

## The Bible as a corpus

### Current and potential uses of the corpus

As we mentioned in the previous section, most parallel corpora are created for SMT training purposes. While the relatively small size of the present corpus makes it rather unsuitable for the creation of full-scale SMT systems across the 4,950 language pairs, we believe that it can be used to tune the probability distributions of an existing SMT system for a phylogenetically similar language. Alternatively it can be used as a source of bi/multi-lingual dictionaries in emergency situations where human translators or other linguistic resources are not available [e.g. the earthquakes in Haiti (Lewis 2010) or Japan (Neubig et al. 2011)].

Steinberger et al. (2013) list a number of potential uses of parallel corpora in NLP. These include: annotation projection for co-reference resolution, discourse analysis; checking translation consistency automatically; testing and benchmarking alignment software (for sentences, words, etc.); producing multilingual lexical and semantic resources such as dictionaries and ontologies; annotation projection across languages for Named Entity Recognition (NER, Ehrmann et al. 2011), sentiment analysis (Steinberger et al. 2011), multi-document summarization (Turchi et al. 2010); cross-lingual plagiarism detection (Potthast et al. 2011); multilingual and cross-lingual document classification (Wei et al. 2008); creation of multilingual semantic space in Lexical Semantic Analysis (LSA, Landauer and Littman 1991) and Kernel Canonical Correlation Analysis (KCCA, Vinokourov et al. 2002). We believe that, despite some disadvantages (e.g. the lack of modern named entities and other issues discussed in Sect. 2.3), the Bible is an excellent resource for NLP, especially for low-resource languages.

Multilingual corpora are also ideal for typological or comparative language analysis, especially when a large number of languages can be collected. Indeed the present corpus has already been used for cross-linguistic induction and comparison of syntactic categories (Christodoulopoulos 2013, pp. 143–159). Similarly, we believe that parallel corpora can be invaluable to the whole area of Digital Humanities (e.g. Dipper and Schultz-Balluff 2013).

### Advantages

There are a number of advantages to using the Bible as a corpus. Not only has it been translated into numerous languages; it has also been translated into a much more diverse set of languages than any other book. This is mostly due to the efforts of missionary linguists such as the Summer Institute of Linguistics (SIL, Brend and Pike 1977) that combine anthropological and linguistic research with missionary expeditions in remote locations and, as a result, produce Bible translations.

Another advantage of the Bible is the size of the text. The complete canonical 66 books contain around 800k words in English. This might seem small compared to modern (parallel) corpora—like, for instance, the Canadian Hansards corpus (Germann 2001) with ~19 M words, and the Europarl (~60 M words on average per language); however it is much bigger than any single work of literature: for instance, the size of the average fiction novel is about 100k words, while ‘Pinocchio’ is ~45 k.

The Bible also is unique as a text since every verse is uniquely identified by a book, chapter and verse number. This allows for an automatic, unambiguous alignment at the verse level across every language (with minor exceptions that will be discussed in Sect. 3).

A final advantage is that the Bible translations collected here are either public domain, or—as in the case of the King James Version—free to use for research purposes.^3^

### Potential issues

#### Translation methods

As with every translation work, one important question concerns the style and fidelity of translation. There are two competing translation methods: *word-for-word* (or formal equivalence), in which the literal meaning of each words as well as the syntactic structure is preserved where possible; and *sense-for-sense* translation (or dynamic equivalence), in which the ‘spirit’ or emotional effect of the text is kept. The former method is more appropriate for the type of analysis required here and has been put forward as the preferred method by the Catholic Church (2001), among others. However, some of the translation guides used by the missionary linguists follow the latter method. For instance Nida and Taber (1969) provide a theoretical framework as well as a set of principles for Bible translations, in which they advise:

- Content is to have priority over style.
- Contextual consistency is to have priority over verbal consistency.
- Long, involved sentences are to be broken up on the basis of receptor-language usage.
- Nouns expressing events should be changed to verbs whenever the results would be more in keeping with receptor-language usage. (Nida and Taber 1969, p. 182)

This does not imply that every Bible translator has followed these principles, but given that the goal of the missionary linguists was to convey the message of the Bible, it makes sense that they would choose a more content-sensitive approach to their translations.

Finally, we should keep in mind that it is not always desirable to have a formally equivalent translation^4^: for instance in MT, when translating the title of Stig Larsson’s third book, a translation system should return “The Girl Who Kicked the Hornet’s Nest” instead of the literal translation of the Swedish “Luftslottet som sprängdes” which would be “The air castle that was exploded”. However, from a computational linguistics perspective, it is usually more helpful to have formally equivalent translations.

#### Other issues

A major issue that is relevant to the use of the Bible as a parallel corpus is the writing style; in particular, the use of antiquated language. This is especially problematic in languages (mostly Western European) where Bible translations were created hundreds of years in the past. Even if modern translations exist, often the editors would choose a more archaic style of writing to match the earlier text and to give the appropriate gravity to the material. Some exceptions exist, at least in English. As Resnik et al. (1999) showed, the New International Version (NIV) covers a significant variety of present-day terms as found in Longman Dictionary of Contemporary English (LDOCE, Proctor 1978) and in the Brown Corpus (Francis 1964).

For many translations, it is an open question whether the writing style of the Bible is representative of present-day language, but given the limited availability of written sources in some languages, and the breadth of available translations, the Bible corpus represents the best resource for cross-linguistic analysis. Indeed there have been a number of projects that used Bible translations as either a primary or secondary source of material (Resnik et al. 1999; Yarowsky and Ngai 2001; Wierzbicka 2001; Kanungo et al. 2005).

A final limiting factor is the fact that the alignment information is limited to verses (rather than sentences as is the case in the JRC-Acquis corpus for instance). While it is often the case that a verse corresponds to a whole sentence, there are verses that span more than two sentences, or are limited to sub-sentence phrases. The exact number varies depending on what is considered to be sentence-final punctuation. When counting only ‘.’ and ‘?’, out of the ~30,000 verses, only 4,000 contain multiple sentences. However, this number increases to 10,000 if we include ‘;’ and more than half the verses if we add ‘:’ as a sentence-final marker. To make things worse, as we can see in the following example, different translations use different punctuation schemas which means that they contain significantly different numbers of sentences.

1.       a.  [A ka ki te Atua], [Kia marama]: [na ka marama]                   (Maori)
        b. [Gu$\eth$ sag$\eth$i]:  [“Ver$\eth$i ljós!”]  [Og þa$\eth$ var$\eth$ ljós].                   (Icelandic)
        c. [Dio disse]: [≪Sia la luce!≫]. [E la luce fu]                          (Italian)
        d. [dixitque Deus] [fiat lux] [et facta est lux]                                (Latin)
        e.  [And God said], [Let there be light]: [and there was light].      (English)

## Acquiring and converting source material

### Corpus collection

Despite the great number of translations, many Bible translations exist only in audio form. This is reasonable, since some of the translated languages exist only in verbal form, and even if an alphabet exists, most speakers of that language may be illiterate. Furthermore, even when textual resources have been available for years, electronic copies are hard to obtain. This means that there is a limited availability of machine-readable bibles online. In English, for instance, one of the most widespread Bibles, the King James Version, is not made available in electronic form by the official licensing body in Scotland (the Scottish Bible Board) even though the text is free to use for research purposes. Instead, we have had to rely on third-party sources, like the ones mentioned in the next paragraph. When multiple versions of the Bible were available—since the aim of this project was breadth instead of depth—we opted for a single translation, usually the oldest available one (e.g. the King James Version for English). We believe that this will lead to a more coherent corpus, as older translations tend to be more literal, but we acknowledge that this brings up the issue of diachronic language change. As discussed in Sect. 1, this problem is not as severe as initially perceived; however we are also open to the idea of adding multiple versions of the same language in the future.

**Fig. 1:**
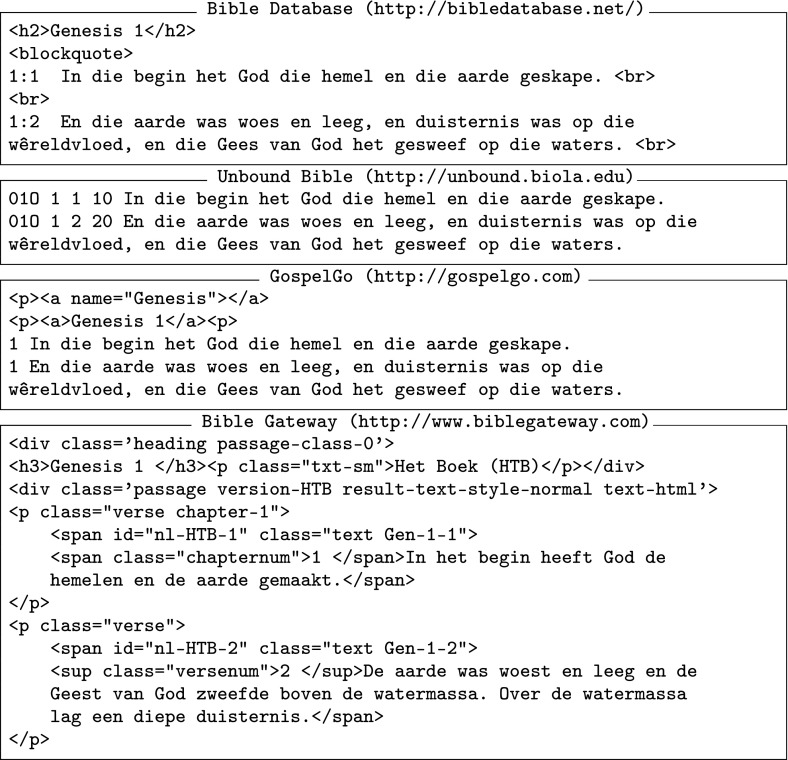
Different Bible online versions of Gen:1–2 in Afrikaans (last box in the figure is in Dutch)

**Fig. 2:**
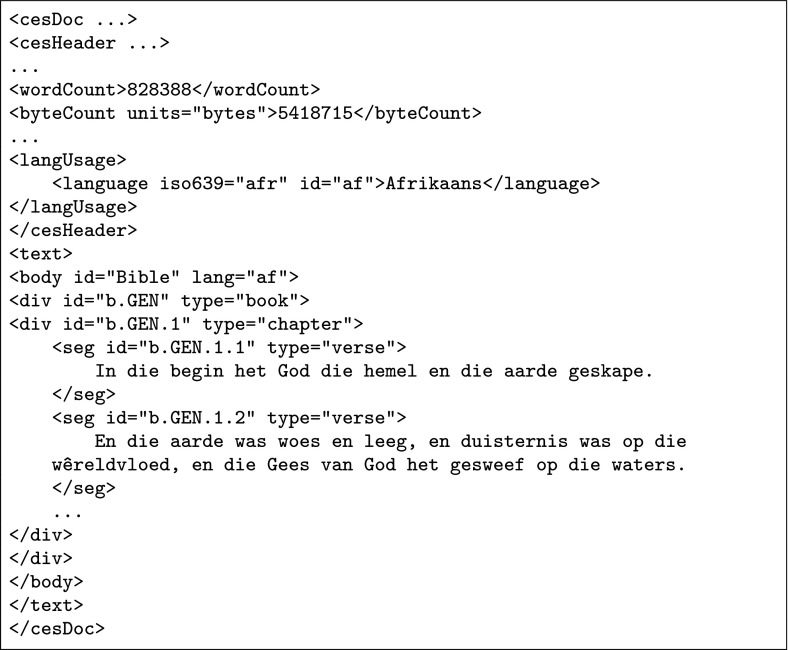
Level 1 CES annotation

There are a few websites that offer access to public domain, machine-readable versions of the Bible in multiple languages. The four main sources used here were the *Bible Database*, the *Unbound Bible*, *GospelGo* and the *Bible Gateway* websites. Each one offered the Bible in different formats, some containing HTML and others plain text. Figure 1 presents a comparison of the different versions.

In order to unify all the different styles of annotation under a well-defined universal format, we followed Resnik et al. (1999) in using the Corpus Encoding Standard (CES, Ide 1998), conforming to the level 1 annotation guidelines. Practically, this means that each Bible was formatted as an XML file, containing nested <div> elements corresponding to books and chapters, and <seg> elements that corresponded to verses. Each of the verses was marked with a serial ID. Figure 2 shows the same two verses of Fig. 1 as formatted by custom scripts.

### Conversion problems

The most common issue we encountered when converting and formatting our corpus was the inconsistency in the formatting of the online sources. Some of the more common ones included incorrect HTML: unclosed <span> or <p> tags, inconsistent use of capitalisation (e.g. <SPAN> Verse Text </span>); errors in verse numbering (e.g. “missing” verses were actually included in previous or subsequent verses marked by text instead of HTML tags); character rendering errors (e.g. ž in Croatian rendered as ?); missing characters (e.g. final character in each verse of the Thai and Latin translations). In most cases the errors were systematic and could be corrected semi-automatically; in other cases (like the missing characters) we had to find multiple sources of the same translation. If neither option was available, the errors were left in the final version of the corpus. Overall, the whole process took about two-to-three person/months.

Finally, when dealing with machine-readable multilingual texts, character encoding can cause difficulties. This is especially true for languages that do not have a strong international presence and the need to adopt an encoding standard is low. However, we did not encounter such problems during the creation of this corpus; all languages included in the corpus have been encoded using the Universal Character Set (UCS, Allen et al. 2012), specifically the UCS Transformation Format-8-bit (UTF-8).

## Parallel corpus information

The full corpus contains 100 languages from across the world (see Table 1 for the names of the languages). As Table 2 shows, the majority are non-Indo-European languages and 39 of the languages are spoken by fewer than 1 million speakers.

**Fig. 3:**
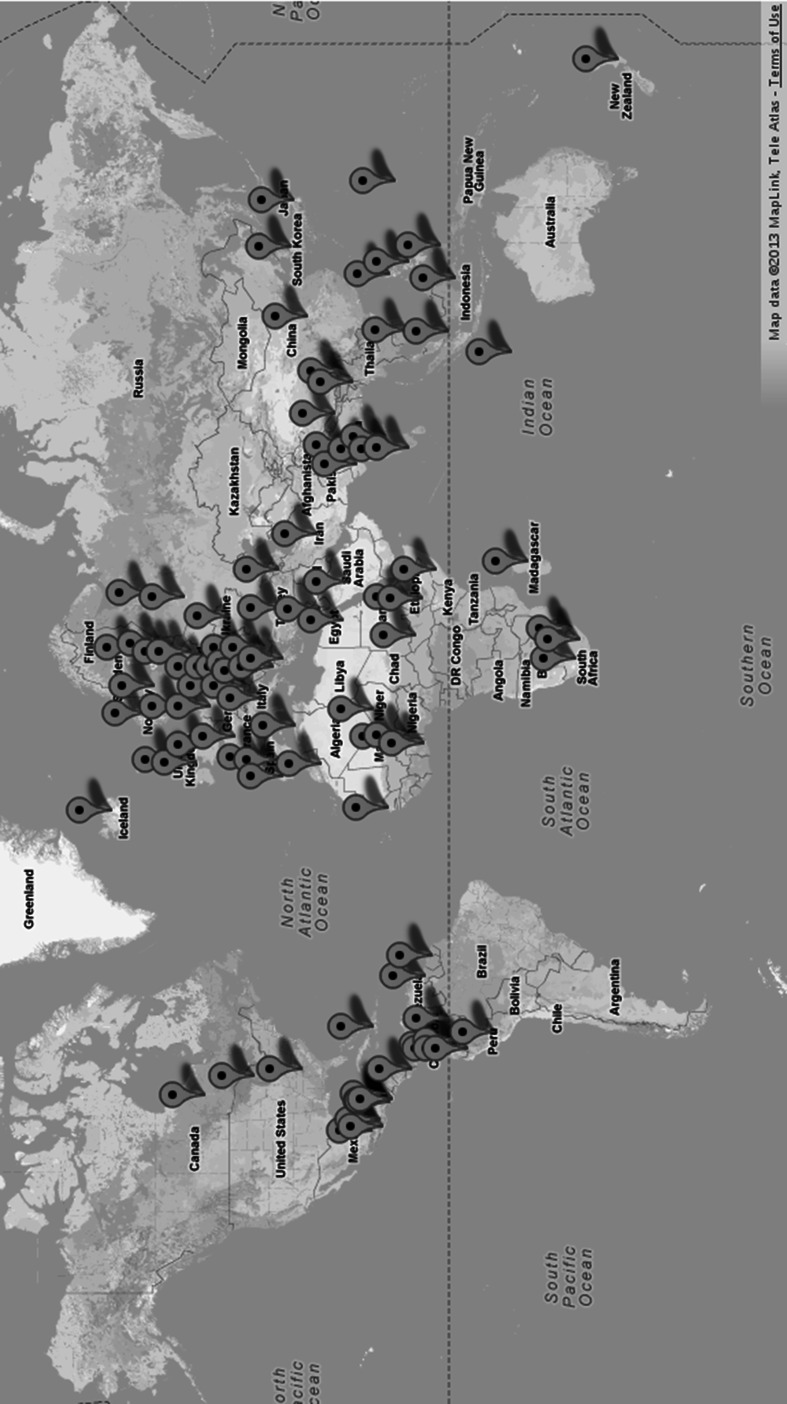
A map of the distribution of languages in the Bible Corpus. Each pin represents the country or territory where the language originates or is primarily used (e.g. there is only one pin for English in Britain). Location coordinates were acquired from Dryer and Haspelmath (2013)

Figure 3 presents a geographical distribution of the languages (data from Dryer and Haspelmath 2013) that cover almost all the continents, and Appendix A contains detailed linguistic information about every language as well as the approximate date of translation (data from Lewis et al. 2014).

**Table 1:** Languages in the Bible Corpus

Achuar-Shiwiar	Gaelic (Scottish)^†^	**Polish**
**Afrikaans**	Galela	**Portuguese**
Aguaruna	**German**	Potawatomi^†^
Akawaio	**Greek**	**Q’eqchi’**
**Albanian**	Gujarati	Quichua
Amharic	**Haitian Creole**	Romani
Amuzgo	**Hebrew**	**Romanian**
**Arabic**	**Hindi**	**Russian**
Armenian^†^	**Hungarian**	**Serbian**
Aukan	**Icelandic**	Shuar (Jivaro)
Barasana-Eduria	**Indonesian**	**Slovak**
Basque	**Italian**	**Slovene**
**Bulgarian**	Jakalteko	**Somali**
Cabécar	**Japanese**	**Spanish**
Cakchiquel	K’iche’	Swahili
Campa (Asháninka)	Kabyle	**Swedish**
Camsá	**Kannada**	Syriac
**Cebuano**	**Korean**	Tachelhit
Chamorro^†^	**Latin**	**Tagalog**
Cherokee	Latvian	Tamajaq (Tuareg)^†^
Chinantec (Quiotepec)	**Lithuanian**	Telugu
**Chinese**	Lukpa	**Thai**
Coptic	**Malagasy**	**Turkish**
**Croatian**	**Malayalam**	Ukranian
**Czech**	Mam	Uma
**Danish**	Manx^†^	Uspanteco
Dinka	**Maori**	**Vietnamese**
**English**	**Marathi**	Wolaytta
**Esperanto**	**Myanmar (Burmese)**	Wolof
**Estonian**	Nahuatl (Tetelcingo)	**Xhosa**
Ewe	**Nepali**	**Zarma**
**Farsi** (Persian)	**Norwegian**	Zulu
**Finnish**	Ojibwa	
**French**	Paite (Chin)	

The languages containing the full Bible text are in bold. Most of the remaining languages contain the New Testament part of the Bible only (languages marked with ^†^ contain smaller parts)

**Table 2:** Bible Corpus language information

	# Languages
Non-Latin script	28
<1M speakers	39
Non-Indo-European	66
Partial Texts	45

**Fig. 4:**
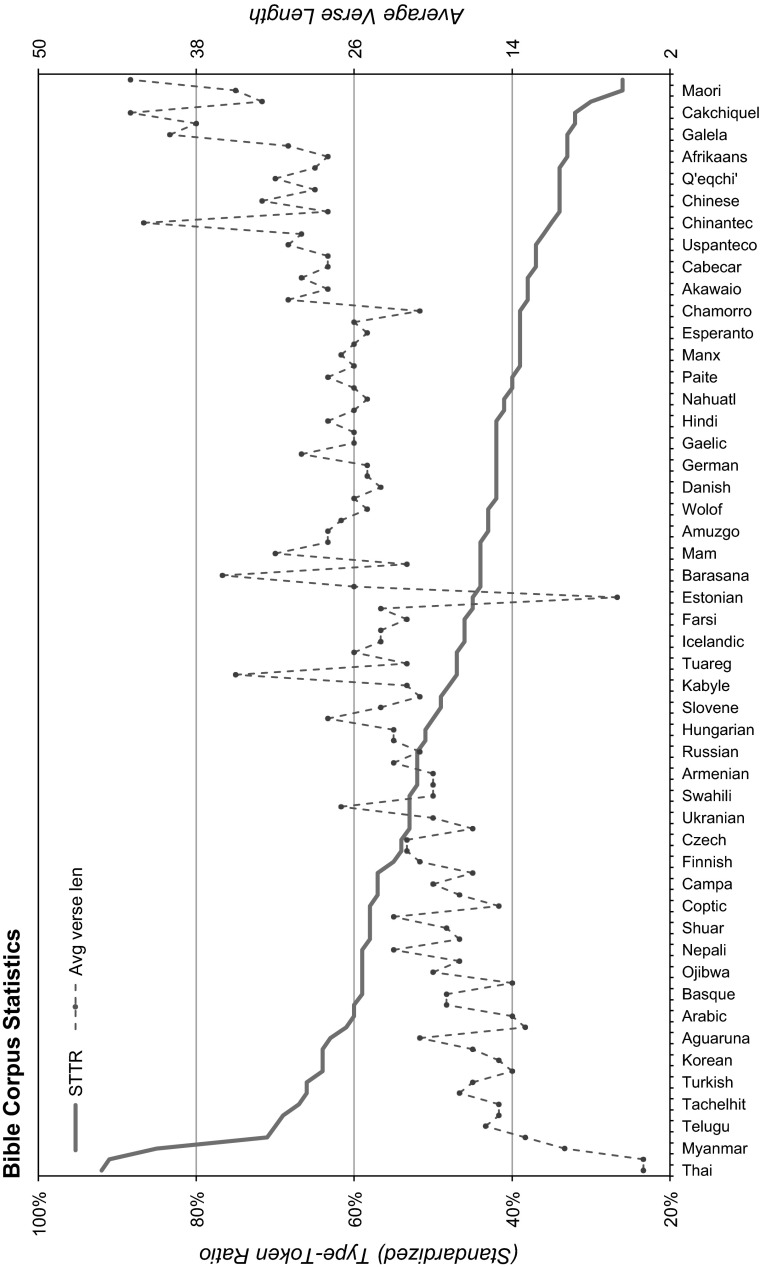
Standardized type-token ratio and average verse length information for each of the languages in the corpus

Table 3 contains statistics about the average size and variability of the lexicon of the whole corpus: we include total number of tokens,^5^ standardised type-token ratio (STTR), average verse length and the percentage of the corpus covered by the 1,000 most frequent words. We also present STTR and average verse length information for each individual language in Fig. 4.

**Table 3:** Bible Corpus statistics

Corpus	# Tokens	STTR (%)	Length	SD	Top-1,000 cover (%)
Bible-avg	432,691	48.59	23.82	7.46	73.80
Bible-eng	789,635	34.42	28.35	12.58	88.69
WSJ	1,173,760	48.89	24.92	12.57	74.11
1984-novel	122,644	47.56	19.99	15.20	81.89
CHILDES	366,509	32.17	4.45	3.04	93.60

STTR is standardised type-token ratio; length refers to the average/standard deviation number of tokens in each verse (or sentence for the other corpora). Bible-avg is the (macro) average over all the languages in the corpus; WSJ is the Wall Street Journal portion of the Penn Treebank (Marcus et al. 1993); George Orwell’s 1984 novel is part of the MULTEXT-East corpus (Erjavec 2004); CHILDES (MacWhinney 2000) is a corpus of child-directed speech utterances

In order to normalise over the overall size of each corpus, we computed STTR by calculating a macro-average over successive measurements of the token-type ratio (# unique word types/# all tokens) of a fixed amount of tokens. This fixed amount corresponded to the smallest number of tokens (678 tokens in Gaelic). We also include the specific numbers for the English translation as well as number from other corpora for comparison: the Wall Street Journal (WSJ) portion of the Penn Treebank (Marcus et al. 1993), George Orwell’s 1984 novel and a corpus of child-directed speech (CHILDES; MacWhinney 2000).

We can see that although the average type-token ratio of the corpus is close to that of both WSJ and 1984, the English translation has far fewer unique word types. Following Resnik et al. (1999), we also compared the vocabulary of the English translation with that of the other English corpora we had available. As Table 4 shows, even though the language King James’ Version of the Bible is more archaic than the New International Version (used in Resnik et al’s comparisons), it still covers a significant portion of the most frequent words in all three corpora.

**Table 4:** English Bible coverage of the most frequent words in three corpora

corpus$_{topN}$	Coverage	Percentage	Missing words cover (%)
WSJ$_{500}$	347	69.40	8.03
1984$_{500}$	423	86.40	3.42
CHILDES$_{500}$	401	80.20	6.75
WSJ$_{1000}$	403	59.70	12.18
1984$_{1000}$	762	76.20	5.95
CHILDES$_{1000}$	709	70.90	8.85

For a qualitative view of the omissions, we present the 10 most frequent words of each corpus that are missing from the KJV Bible: From the WSJ corpus the words are mostly market-related: *million, Mr, says, billion, Corp, inc, shares, president, Co, sales*; in the case of 1984 they are words related to the story of the novel: *winston, party, o’brien, telescreen, big, human, don’t, merely, oceania, minutes*; and finally from CHILDES they are mostly informal, spoken constructions: *yeah, does, huh, alright, ya, okay, gonna, mhm, big, baby*. It is interesting to note that words like ‘big’ and ‘human’ are in these lists. This is due to stylistic differences as well as actual diachronic language changes.

## Remaining problems in the parallel corpus

As Table 2 shows, 45 out of the 100 languages contain only partial texts. In most cases this means that only the New Testament was available for that language, but in a few cases even less text exists. This means that if we want to use all 100 languages, we are limited to the smallest amount of text contained in any of them.

A further problem is the fact that not all the canonical verses (i.e. verses that appear in the original Greek, Hebrew and Aramaic) are present even in the official translations. One possible explanation is that the missing verses are contained in the verses that come before, or after them. This is a reasonable assumption, since in some languages it might not be easy to follow the sentence structure of the original text (e.g. a sentence that is split across two verses). For instance, in the Turkish text, verses no. 2 and 3 of chapter 7 of the Book of Genesis are combined:^6^

GEN.7.2: Yeryüzünde soyları tükenmesin diye, yanına temiz sayılan hayvanlardan erkek ve dişi olmak üzere yedişer çift, kirli sayılan hayvanlardan birer çift, kuşlardan yedişer çift al.

[Gloss] Extinction on earth, lest next clean counted seven pairs of animals, including male and female, a pair of unclean animals, birds take seven pairs.

$$\_\_\_\_\_\_\_\_\_\_\_\_\_\_\_$$

GEN.7.2: Of every clean beast thou shalt take to thee by sevens, the male and his female: and of beasts that are not clean by two, the male and his female.

GEN.7.3: Of fowls also of the air by sevens, the male and the female; to keep seed alive upon the face of all the earth.

In fact, the most commonly missing verse in the New Testament is 2 Corinthians 13:14 (missing from 33 languages where the median is 2) which is a known versification difference.^7^

Of course an alternative explanation would be that some verses were completely omitted, either intentionally or unintentionally (see footnote 7). This seems to be the case in the Swedish translation, where verse no. 29 of chapter 28 in the Book of Acts is missing and the text does not appear in either verse no. 28 or 30.

ACT.28.28: Det mån I därför veta: till hedningarna bar denna Guds frälsning blivit sänd; de skola ock akta därpå.

[Gloss] Be it known therefore know the pagans wore this salvation of God is sent; they will also hearken.

ACT.28.30: I två hela år bodde han sedan kvar i en bostad som han själv hade hyrt. Och alla som kommo till honom tog han emot;

[Gloss] For two whole years he lived then left in a residence that he had rented. And everyone who came to him, he received;

$$\_\_\_\_\_\_\_\_\_\_\_\_\_\_\_$$

ACT.28.28: Be it known therefore unto you, that the salvation of God is sent unto the Gentiles, and that they will hear it.

ACT.28.29: And when he had said these words, the Jews departed, and had great reasoning among themselves.

ACT.28.30: And Paul dwelt two whole years in his own hired house, and received all that came in unto him,

There are cases where multiple verses are omitted like, for instance in the Marathi translation: the first verse of the first chapter in the Book of Ezekiel is verse no. 5, with no information about the previous four verses. However neither the single nor the continuous omissions are very frequent. When we examine all the translations that contain the full text, there are on average 19.38 single verse omissions and 9.69 continuous ones. This amounts to 0.06 and 0.03 % of the total number of verses.

One way to deal with these omissions would be to ignore verses in all languages where text is missing even in one of the languages in the corpus.^8^ Even with this drastic strategy, the overall loss of text across languages may be found to be tolerable: on average, each full bible translation contains about 643,000 words: after the elimination of all non-shared verses, we found the average word count to be about 549,000—only a 14.7 % reduction.

It should be noted, however, that the corpus presented here contains all available verses in all the languages (each with a unique ID as shown in Fig. 2), meaning that, depending on which subset of languages chosen, the limitations described above might not apply. Researchers are encouraged to choose their own methods to deal with these occasional unilateral omissions, whose detection is a precondition to finer-grain sentence- and word–level alignment of the kind proposed by Abney and Bird (2010, 2011).

## Conclusion

This paper described the creation of a massively parallel corpus, consisting of translations of the Bible in 100 languages. We discussed some of the problems arising from the nature of the texts as well as the process of gathering and annotating the online material. The texts in each language were aligned up to the level of verse in compliance with the CES guidelines. While a few more Bible translations exist in a machine-readable form (as well as a number of different translations for some languages), we believe this set of 100 languages is significantly large for an initial release. We expect to add more languages if the resource is used, and we encourage such additions by other researchers. We have released code to allow users to add more languages to the corpus as well as process the existing ones, and together with the annotated XML files, they are published under a Creative Commons license and can be found at the following address: http://groups.inf.ed.ac.uk/ccg/corpora.html.

## Appendix A

See Table 5.

**Table 5:** Linguistic details and available parts of the Bible corpus with approximate translation date (linguistic data and translation dates from Lewis et al. 2014)

ISO 639-3	Language	Family	Genus	Subgenus	Speakers	Script	Full	Parts	Year
acu	Achuar-Shiwiar	Jivaroan			5,000	Latin	N	NT	1981
afr	Afrikaans	Indo-European	Germanic	West	5,000,000	Latin	Y		1953
agr	Aguaruna	Jivaroan			38,300	Latin	N	NT	1973
ake	Akawaio	Carib	Northern	East-West Guiana	4,500	Latin	N	NT	2010
als	Albanian	Indo-European	Albanian	Tosk	3,000,000	Latin	Y		1993
amh	Amharic	Afro-Asiatic	Semitic	South	17,500,000	Ethiopic	N	NT	1840
amu	Amuzgo	Oto-Manguean	Amuzgoan		23,000	Latin	N	NT	1973
arb	Arabic	Afro-Asiatic	Semitic	Central	206,000,000	Arabic	Y		1865
hye	Armenian	Indo-European	Armenian		64,00,000	Armenian	N	Parts	1883
djk	Aukan	Creole	English based	Atlantic	15,500	Latin	N	NT	1999
bsn	Barasana-Eduria	Tucanoan	Eastern Tucanoan	Central	1,890	Latin	N	NT	2001
eus	Basque	Basque			700,000	Latin	N	NT	1855
bul	Bulgarian	Indo-European	Slavic	South	9,000,000	Cyrillic	Y		1864
cjp	Cabécar	Chibchan	Talamanca		8,840	Latin	N	NT	1993
cak	Cakchiquel	Mayan	Quichean	Greater Quichean	132,000	Latin	N	NT	1931
cni	Campa (Asháninka)	Arawakan	Maipuran	Southern Maipuran	26,100	Latin	N	NT	1972
kbh	Camsá	Equatorial (?)			4,770	Latin	N	NT	1990
ceb	Cebuano	Austronesian	Malayo-Polynesian	Phillipine	15,800,000	Latin	Y		1917
cha	Chamorro	Austronesian	Malayo-Polynesian	Chamorro	92,000	Latin	N	Parts	2007
chr	Cherokee	Iroquoian	Southern Iroquoian		16,400	Cherokee	N	NT	1850
chq	Chinantec (Quiotepec)	Oto-Manguean	Chinantecan		8,000	Latin	N	NT	1983
cmn	Chinese	Sino-Tibetan	Sinitic	Chinese	840,000,000	Chinese	Y		1874
cop	Coptic	Afro-Asiatic	Egyptian		Extinct	Coptic	N	NT	1716
hrv	Croatian	Indo-European	Slavic	South	5,500,000	Latin	Y		1831
ces	Czech	Indo-European	Slavic	West	9,500,000	Latin	Y		1380
dan	Danish	Indo-European	Germanic	North	5,500,000	Latin	Y		1550
dik	Dinka	Nilo-Saharan	Eastern Sudanic	Nilotic	450,000	Latin	N	NT	2006
eng	English	Indo-European	Germanic	West	328,000,000	Latin	Y		1611
epo	Esperanto	Constructed			1,000	Latin	Y		1900
est	Estonian	Uralic	Finno-Ugric	Finno-Permic	1,000,000	Latin	Y		1739
ewe	Ewe	Niger-Congo	Atlantic-Congo	Volta-Congo	2,250,000	Latin	N	NT	1911
pes	Farsi (Persian)	Indo-European	Indo-Iranian	Iranian	22,000,000	Arabic	Y		1838
fin	Finnish	Uralic	Finno-Ugric	Finno-Permic	5,000,000	Latin	Y		1776
fra	French	Indo-European	Italic	Romance	58,000,000	Latin	Y		1776
gla	Gaelic (Scottish)	Indo-European	Celtic	Insular	67,000	Latin	N	Parts	1801
gbi	Galela	West Papuan	North Halmahera	Galela-Loloda	79,000	Latin	N	NT	2002
deu	German	Indo-European	Germanic	West	90,300,000	Latin	Y		1545
ell	Greek	Indo-European	Greek	Attic	13,000,000	Greek	Y		1840
guj	Gujarati	Indo-European	Indo-Iranian	Indo-Aryan	45,500,000	Gujarati	N	NT	1823
hat	Haitian Creole	Creole			7,700,000	Latin	Y		1985
heb	Hebrew	Afro-Asiatic	Semitic	Central	5,300,000	Hebrew	Y		1599
hin	Hindi	Indo-European	Indo-Iranian	Indo-Aryan	180,000,000	Devanagari	Y		1818
hun	Hungarian	Uralic	Finno-Ugric	Ugric	12,500,000	Latin	Y		1590
isl	Icelandic	Indo-European	Germanic	North	230,000	Ethiopic	Y		1863
ind	Indonesian	Austronesian	Malayo-Polynesian	Malayo-Sumbawan	23,100,000	Latin	Y		1974
ita	Italian	Indo-European	Italic	Romance	61,700,000	Latin	Y		1649
jai	Jakalteko	Mayan	Kanjobalan-Chujean	Kanjobalan	77,700	Latin	N	NT	1979
jpn	Japanese	Japonic			122,000,000	Kanjii	Y		1883
quc	K’iche’	Mayan	Quichean-Mamean	Greater Quichean	1,900,000	Latin	N	NT	1995
kab	Kabyle	Afro-Asiatic	Berber	Northern	3,100,000	Latin	N	NT	2011
kan	Kannada	Dravidian	Southern	Tamil-Kannada	35,300,000	Kannada	Y		1831
kor	Korean	Altaic(?)			66,300,000	Hangul	Y		1911
lat	Latin	Indo-European	Italic	Latino-Faliscan	Extinct	Latin	Y		400
lav	Latvian	Indo-European	Baltic	Eastern	1,500,000	Latin	N	NT	1689
lit	Lithuanian	Indo-European	Baltic	Eastern	3,100,000	Latin	Y		1735
dop	Lukpa	Niger-Congo	Atlantic-Congo	Volta-Congo	50,000	Latin	N	NT	2009
plt	Malagasy	Austronesian	Malayo-Polynesian	Greater Barito	7,520,000	Latin	Y		1835
mal	Malayalam	Dravidian	Southern	Tamil-Kannada	35,400,000	Malayalam	Y		1841
mam	Mam	Mayan	Quichean-Mamean	Greater Mamean	200,000	Latin	N	NT	1993
glv	Manx	Indo-European	Celtic	Insular	77,000	Latin	N	Parts	1773
mri	Maori	Austronesian	Malayo-Polynesian	Central-Eastern	60,000	Latin	Y		1858
mar	Marathi	Indo-European	Indo-Iranian	Indo-Aryan	68,000,000	Devanagari	Y		1821
mya	Myanmar (Burmese)	Sino-Tibetan	Tibeto-Burman	Lolo-Burmese	32,300,000	Myanmar	Y		1835
nhg	Nahuatl (Tetelcingo)	Uto-Aztecan	Southern Uto-Aztecan	Aztecan	3,500	Latin	N	NT	1980
nep	Nepali	Indo-European	Indo-Iranian	Indo-Aryan	11,100,000	Devanagari	Y		1914
nor	Norwegian	Indo-European	Germanic	North	4,600,000	Latin	Y		1904
ojb	Ojibwa	Algic	Algonquian	Central	20,000	Aboriginal Syllabics	N	NT	1988
pck	Paite (Chin)	Sino-Tibetan	Tibeto-Burman	Kuki-Chin-Naga	78,800	Latin	Y		1971
pol	Polish	Indo-European	Slavic	West	36,600,000	Latin	Y		1975
por	Portuguese	Indo-European	Italic	Romance	178,000,000	Latin	Y		1751
pot	Potawatomi	Algic	Algonquian	Central	1,300,000	Latin	N	Parts	1844
kek	Q’eqchi’	Mayan	Quichean-Mamean	Greater Quichean	400,000	Latin	Y		1988
quw	Quichua	Quechuan	Quechua II	B	20,000	Latin	N	NT	1972
rmn	Romani	Indo-European	Indo-Iranian	Indo-Aryan	710,000	Latin	N	NT	2008
ron	Romanian	Indo-European	Italic	Romance	23,400,000	Latin	Y		1928
rus	Russian	Indo-European	Slavic	East	143,000,000	Cyrillic	Y		1876
srp	Serbian	Indo-European	Slavic	South	7,000,000	Latin	Y		1804
jiv	Shuar (Jivaro)	Jivaroan			46,700	Latin	N	NT	2010
slk	Slovak	Indo-European	Slavic	West	4,610,000	Latin	Y		1832
slv	Slovene	Indo-European	Slavic	South	1,730,000	Latin	Y		1584
som	Somali	Afro-Asiatic	Cushitic	East	8,340,000	Latin	Y		1979
spa	Spanish	Indo-European	Italic	Romance	328,000,000	Latin	Y		1569
swh	Swahili	Niger-Congo	Atlantic-Congo	Volta-Congo	788,000	Latin	N	NT	1891
swe	Swedish	Indo-European	Germanic	North	8,300,000	Latin	Y		1917
arc	Syriac	Afro-Asiatic	Semitic	Central	Extinct	Syriac	N	NT	464
shi	Tachelhit	Afro-Asiatic	Berber	Northern	3,000,000	Arabic	N	NT	2010
tgl	Tagalog	Austronesian	Malayo-Polynesian	Phillipine	23,900,000	Latin	Y		1905
ttq	Tamajaq (Tuareg)	Afro-Asiatic	Berber	Tamasheq	640,000	Latin	N	Parts	1979
tel	Telugu	Dravidian	South-Central	Telugu	69,600,000	Telugu	Y		1854
tha	Thai	Tai-Kadai	Kam-Tai	Be-Tai	20,300,000	Thai	Y		1883
tur	Turkish	Altaic	Turkic	Southern	50,000,000	Latin	Y		1827
ukr	Ukranian	Indo-European	Slavic	East	37,000,000	Cyrillic	N	NT	1903
ppk	Uma	Austronesian	Malayo-Polynesian	Celebic	20,000	Latin	N	NT	1996
usp	Uspanteco	Mayan	Quichean-Mamean	Greater Quichean	3,000	Latin	N	NT	1999
vie	Vietnamese	Austro-Asiatic	Mon-Khmer	Viet-Muong	68,600,000	Latin	Y		1934
wal	Wolaytta	Afro-Asiatic	Omotic	North	1,230,000	Ethiopic	N	NT	1981
wol	Wolof	Niger-Congo	Atlantic-Congo	Atlantic	4,000,000	Latin	N	NT	1988
xho	Xhosa	Niger-Congo	Atlantic-Congo	Volta-Congo	7,800,000	Latin	Y		1859
dje	Zarma	Nilo-Saharan	Songhai	Southern	2,350,000	Latin	Y		1990
zul	Zulu	Niger-Congo	Atlantic-Congo	Volta-Congo	9,980,000	Latin	N	NT	1883

## Footnotes

1 There are other, non-parallel but *comparable* corpora that exist in multiple languages (like Wikipedia with more than 10,000 articles in 121 languages) but their use is limited to a few approaches (e.g. Cohen et al. (2011), mentioned above).

2 The number of unique language pairs among *n* languages irrespective of the order in each pair is $${\left( \begin{array}{l}{n}\\ {2}\end{array}\right) } = \frac{n!}{2!(n-2)!}$$

3 We have tried to ensure that all translations we have used here are indeed free for research purposes and will comply with any restrictions that we have inadvertently overlooked.

4 Thanks to the anonymous reviewer for pointing us to this example.

5 Calculating the number of tokens is not always straightforward, especially in languages/scripts where words are not tokenised by spaces. In the present study we only perform white-space (and punctuation) tokenisation, which means that the numbers for specific languages (e.g. Japanese, Thai) are going to be misleading.

6 Glosses are provided using Google translate.

7 There are other known omissions of verses whose authenticity has been doubted; see http://en.wikipedia.org/wiki/List_of_Bible_verses_not_included_in_modern_translations for a list. After examining the number of languages where each verse is missing, the verses listed here are indeed missing in an above-average number of languages: most of them are in the 0.6 percentile and some in the 0.9 percentile (e.g. MAR.9.46, ACT.8.37). Thanks to an anonymous reviewer for pointing us to these cases.

8 The alternative approach would be to use a simple heuristic where if a verse is missing in any language, then its contents in all the other languages are merged with the previous verse. However, since there are no guarantees that the text is indeed present in the previous (or the next) verses, the quality of the alignment would be compromised.
